# Body composition and risk of major gynecologic malignancies: Results from the UK Biobank prospective cohort

**DOI:** 10.1002/cam4.3925

**Published:** 2021-06-10

**Authors:** Peng Yun, Bin Xia, Xiao‐hui Tian, Ting Gong, An‐ran Liu, Jin‐qiu Yuan, Fang‐ping Li

**Affiliations:** ^1^ Department of Endocrinology The Seventh Affiliated Hospital of Sun Yat‐sen University Shenzhen China; ^2^ Clinical medicine research center The Seventh Affiliated Hospital of Sun Yat‐sen University Shenzhen China; ^3^ Department of Obstetrics and Gynecology The Seventh Affiliated Hospital of Sun Yat‐sen University Shenzhen China; ^4^ Department of Radiology The Seventh Affiliated Hospital of Sun Yat‐sen University Shenzhen China; ^5^ Department of Nutriology The Seventh Affiliated Hospital of Sun Yat‐sen University Shenzhen China

**Keywords:** body composition, cohort study, gynecological malignancy, UK Biobank

## Abstract

**Objective:**

To evaluate the association between body composition and subsequent risk of the major gynecologic malignancies.

**Methods:**

This is a prospective analysis of participants from the UK Biobank. We measured baseline body composition and confirmed cancer diagnosis through linkage to cancer and death registries. We evaluated hazard ratios (HRs) and confidence interval (CIs) with COX models adjusting for potential confounders.

**Results:**

We document 1430 cases of the top three gynecologic malignancies (uterine corpus cancer 847 cases, ovarian cancer 514 cases, and cervical cancer 69 cases) from 245,084 female participants (75,307 were premenopausal and 169,777 were postmenopausal). For premenopausal women, whole body fat‐free mass (WBFFM) was associated with an increased risk of uterine corpus cancer (Adjusted HR per unit increase 1.04, 95% CI 1.02–1.06). For postmenopausal women, compared with the first quartile, the fourth quartile of WBFFM and whole body fat mass(WBFM) was associated with 2.16 (95% CI 1.49–3.13) times and 1.89 (95% CI 1.31–2.72) times of increased uterine corpus cancer risk, respectively. Regarding the distribution of body fat mass (FM)/fat‐free mass (FFM), FFM distributed in the trunk was associate with increased uterine corpus cancer risk in premenopausal (HR 1.18,95% CI 1.07–1.31) and postmenopausal women (HR 1.13,95% CI 1.09–1.18). Meanwhile, FM/FFM distributed in the limbs present an U‐shaped associations with uterine corpus cancer risk. We did not observe any association between aforementioned body composition indices with ovarian or cervical cancer.

**Conclusion:**

FM is associated with an increased risk of uterine corpus cancer in postmenopausal women. Meanwhile, FFM is found to be a risk factor for uterine corpus cancer in both premenopausal and postmenopausal women. No association of body composition with ovarian or cervical cancer was observed.

## Graphical abstract

The aim of this study is to reveal the relationship between body composition and the risk of gynecological malignancies. In addition to finding that body fat mass(FM) is a risk factor for postmenopausal uterine corpus cancer, we also find that fat‐free mass(FFM) is a stronger risk factor for uterine corpus cancer in both premenopausal and postmenopausal women. No association of body composition with ovarian or cervical cancer was observed.

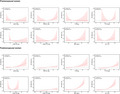

## 1 INTRODUCTION

Cervical, uterine corpus, and ovarian cancers are the most common gynecologic malignancies with high mortality worldwide.^1^ In developed countries, the incidence of uterine corpus and ovarian cancer is far higher than that of cervical cancer.^2^, ^3^ While in developing countries, cervical cancer is the second most commonly diagnosed cancer after breast cancer in female cancers and the third leading cause of cancer‐related death after breast and lung cancers.^2^

The risk factors of gynecologic malignancies vary widely. Uterine corpus cancer, the vast majority of which is endometrial cancer, has been suggested to be in relation to hormone imbalance.^4^ Additionally, overweight/obesity plays an important role: excess body weight alone is estimated to account for about 41% of uterine corpus cancer cases in a UK population‐based cohort study.^5^ Ovarian cancer shares some hormone‐related risk factors with uterine corpus cancer, such as menopausal hormone therapy (HRT) and excess body weight, but whether obesity is a risk factor for ovarian cancer remains controversial.^6^, ^7^ The main risk factor for cervical cancer is chronic infection with human papillomavirus. Obesity has been inconsistently reported to be linked to increased cervical cancer incidence. A recent clinical study shows that overweight and obese women had an increased risk of cervical cancer, which might be own to reduced detection of cervical precancer.^8^

For most previous studies evaluating obesity and risk of gynecological malignancies, traditional anthropometric measurements such as body mass index (BMI), waist circumference (WC), and waist‐to‐hip ratio (WHR) were used as exposure factors. Although these indicators provide simple, cheap, and crude measures of body size, they could neither directly discriminate between fat mass (FM) and fat‐free mass (FFM), nor precisely evaluate the distribution of fat. It has been shown that lower body, upper body, subcutaneous, and visceral fat depots have unique characteristics with regards to fatty acid metabolism. Meanwhile, selective dysregulation of these depots probably plays an important role with the metabolic complications of obesity.^9^ To date, epidemiological evidence for body composition and gynecologic malignancies remain sparse. Limited evidence suggested a positive association between whole body fat mass (WBFM) with increased risk of endometrial cancer among postmenopausal women.^10^ However, this study did not include premenopausal women. The carcinogenic effect of obesity on hormone‐dependent tumors such as endometrial cancer varies with menopausal status.^11^ Therefore, further research is necessary.

UK Biobank is a large prospective study that collected data about body composition and cancer incidence from 0.5 million UK adults.^12^ Based on the UK Biobank dataset, we carry out this prospective analysis to ascertain the relationship between body composition (including whole/trunk/appendicular body FM and FFM) and the risk of major gynecologic malignancies.

## 2 METHODS

### 2.1 Study design and data source

This is a population‐based prospective cohort study based on the UK Biobank dataset. At recruitment in 2006–2010, the participants underwent a range of physical measurements and detailed assessments of health‐related factors. UK Biobank also collected blood, urine, and saliva samples for biochemical analysis. Follow‐up is conducted through linkages to routinely available national datasets. Details of the rationale, design and survey methods for UK Biobank can be found elsewhere.^13^ For the present analysis, we included all participants from UK Biobank who had complete data for FM and FFM. We excluded the participants with a diagnosis of cancer prior to baseline assessment (except for non‐melanoma skin cancer, 10th revisions of international classification of diseases (ICD‐10) C44). UK Biobank was approved by the North West Multicenter Research Ethics Committee (MREC), the Patient Information Advisory Group (PIAG) in England and Wales, and the Community Health Index Advisory Group in Scotland (CHIAG).

### 2.2 Body composition and anthropometry

UK Biobank evaluated FM (kg) and FFM (kg) with electrical bio‐impedance (Tanita BC418MA body composition analyzer) at baseline interview. The whole body as well as site‐specified (truck, leg, and arm) FM/FFM were evaluated. Detailed descriptions of the procedures used to measure body composition can be found on the study website.^14^ UK Biobank also evaluated body composition in 5170 participants using dual‐energy X‐ray absorptiometry(DXA). Assessment of body composition by bio‐impedance and DXA showed high correlation (FFM: r = 0.96, FM: r = 0.86). Trained staff measured standing height using the Seca 202 device (Seca, Hamburg, Germany) and assessed waist/hip circumference with the Wessex non stretchable sprung tape measure (Wessex, United Kingdom). BMI was calculated as the ratio of weight to squared height. We obtained WHR by dividing waist circumference by hip circumference.

### 2.3 Ascertainment of cancer cases

UK Biobank obtains data on cancer diagnosis through the Health & Social Care Information Centre for participants in England and Wales, and the NHS Central Register for participants in Scotland. These registrations recorded the diagnosis of cancer and cancer deaths using ICD‐10 codes. The primary outcome for this study was cervical cancer (C53), uterine corpus cancer (C54‐55), and ovarian cancer (C56).

### 2.4 Data Analysis

We calculated person‐years from the recruitment date to the date of the first diagnosis of cancer, death, or the last date of follow‐up (30 October 2015), whichever came first. We evaluated the risk of the top three gynecologic malignancies by quartiles and as continuous per kilogram (kg) increase in FM/FFM. To compare the ability to predict major gynecologic malignancies (uterine corpus cancer, ovarian cancer, and cervical cancer) across various body composition and anthropometry measures, we evaluated the HRs per standard deviation (SD) increase with Cox regression. To investigate potential nonlinear associations, we fitted restricted cubic splines in Cox regression models. We checked the proportional hazards assumption using Schoenfeld's tests. For covariates with selection of “do not know” and “prefer not to answer,” or with missing covariate data, we included an “unknown/missing” value indicator.

We used Cox regression models, taking age as the underlying timescale, to estimate hazard ratios (HRs) and 95% confidence intervals (CIs) for the association of anthropometric measurements with subsequent gynecologic malignancies risks. We additionally adjusted for ethnic, index of multiple deprivation, smoking status, alcohol consumption, physical activity, fruit and vegetable intake, diabetes, family history of gynecologic malignancies, surgical history (history of uterus, ovaries, and cervical resection), menarche age, oral contraceptives history, reproductive history, and height in the multivariate Cox regression. For the analysis of body composition, we included both FM and FFM in the model to examine their independent effects.

We evaluated potential stratification of age, smoking status, obesity, and family history of cancer on the associations between WBFM/whole body fat‐free mass (WBFFM) and risk of major gynecologic malignancies. The effect of stratification was tested by introducing interaction terms in the regression model. To check the robustness of the primary analysis, a series of sensitivity analyses were performed. First, we excluded cancer diagnosed during the first 2 years of follow‐up to minimize reverse causality. Second, we use the complete‐case analysis to verify the influence of missing data. The statistical analyses were conducted using the SAS (release 9.4; SAS Institute Inc) and R software (version 3.5.0, R Foundation for Statistical Computing, Vienna, Austria).

## 3 RESULTS

This study included a total of 245,009 female participants, of which 75,284 were premenopausal and 169,725 were postmenopausal. The distribution of the study population characteristics by quartiles of FM/FFM is presented in Table 1. For premenopausal and postmenopausal participants, the index of multiple deprivation (IDM) and WC were likely to increase with WBFM. The participants with lower WBFM or WBFFM tended to be the nonsmokers and have a lower rate of hypertension and diabetes.

**TABLE 1:** Characteristics of study participants

Characteristics	Whole body fat mass				Whole body fat free mass			
	Q1	Q2	Q3	Q4	Q1	Q2	Q3	Q4
Postmenopausal								
N	38001	43078	44961	43685	47216	44060	40568	37881
Age, Mean (SD), years	59.6 (5.45)	60.3 (5.31)	60.6 (5.27)	60.2 (5.27)	60.8 (5.32)	60.3 (5.28)	60.0 (5.32)	59.5 (5.33)
Menarche age, Mean (SD), years	13.1 (1.59)	13.0 (1.58)	12.9 (1.61)	12.7 (1.66)	13.1 (1.62)	13.0 (1.59)	12.9 (1.60)	12.8 (1.65)
Menopause age, Mean (SD), years	49.8 (4.71)	50.0 (4.79)	49.9 (5.06)	49.8 (5.29)	49.8 (4.92)	49.9 (4.91)	50.0 (4.93)	49.9 (5.15)
Age at first live birth, Mean (SD), years	25.7 (4.48)	25.2 (4.34)	24.7 (4.31)	24.2 (4.37)	24.9 (4.33)	25.0 (4.36)	25.0 (4.42)	24.8 (4.53)
Null birth number, N (%)	7242 (19.1%)	6485 (15.1%)	6385 (14.2%)	6620 (15.2%)	7639 (16.2%)	6631 (15.0%)	6154 (15.2%)	6308 (16.7%)
White, N (%)	36319 (95.6%)	41296 (95.9%)	43078 (95.8%)	41332 (94.6%)	44164(93.5%)	42454 (96.4%)	39177 (96.6%)	36229 (95.6%)
IDM, Mean (SD)	15.4 (12.4)	15.8 (12.6)	16.8 (13.3)	19.3 (14.7)	16.8 (13.3)	16.3 (12.9)	16.5 (13.1)	18.1 (14.1)
BMI, Mean (SD), kg/m2	22.0 (1.84)	24.9 (1.69)	27.7 (2.01)	33.6 (4.44)	23.9 (3.03)	25.8 (3.32)	27.8 (3.80)	32.4 (5.62)
WC, Mean (SD), cm	72.6 (5.70)	80.0 (5.85)	87.0 (6.50)	99.9 (10.2)	77.4 (8.39)	82.1 (8.97)	86.9 (9.85)	97.3 (12.7)
WHR, Mean (SD)	0.78 (0.06)	0.81 (0.06)	0.83 (0.06)	0.86 (0.07)	0.80 (0.06)	0.81 (0.07)	0.83 (0.07)	0.85 (0.07)
Never smoking, N (%)	22992 (60.5%)	25441 (59.1%)	25643 (57.0%)	24203 (55.4%)	28549 (60.5%)	25609 (58.1%)	23212 (57.2%)	20908 (55.2%)
Never drinking, N (%)	3515 (9.2%)	3593 (8.3%)	4101 (9.1%)	5522 (12.6%)	5108 (10.8%)	3847 (8.7%)	3572 (8.8%)	4204 (11.1%)
MET, Mean (SD), minutes/week	2970 (2690)	2720 (2540)	2490 (2440)	2110 (2270)	2670 (2560)	2660 (2520)	2570 (2490)	2330 (2420)
Fruit and vegetable intake, Mean (SD), portions/day	3.72 (2.87)	3.56 (2.64)	3.41 (2.55)	3.23 (2.50)	3.47 (2.79)	3.48 (2.54)	3.50 (2.57)	3.44 (2.64)
Family history of cancer, N (%)	13976 (36.8%)	16298 (37.8%)	17338 (38.6%)	16869 (38.6%)	17357 (36.8%)	16655 (37.8%)	15800 (38.9%)	14668 (38.7%)
HRT, N (%)	3892 (10.2%)	3837 (8.9%)	3590 (8.0%)	2723 (6.2%)	3641 (7.7%)	3763 (8.5%)	3560 (8.8%)	3078 (8.1%)
Type 2 diabetes, N (%)	1041 (2.7%)	1555 (3.6%)	2236 (5.0%)	4759 (10.9%)	1717 (3.6%)	1713 (3.9%)	2127 (5.2%)	4034 (10.6%)
Hypertension, N (%)	23569 (62.0%)	30162 (70.0%)	34683 (77.1%)	37248 (85.3%)	33494 (70.9%)	31491 (71.5%)	30041 (74.1%)	30636 (80.9%)
Premenopausal								
N	23523	18229	16083	17449	13220	17501	20184	24379
Age, Mean (SD), years	46.2 (3.90)	46.6 (4.16)	46.9 (4.28)	46.9 (4.30)	47.0 (4.49)	46.6 (4.18)	46.5 (4.08)	46.4 (3.99)
Menarche age, Mean (SD), years	13.3 (1.58)	13.1 (1.57)	13.0 (1.61)	12.7 (1.68)	13.2 (1.63)	13.1 (1.59)	13.1 (1.59)	12.8 (1.64)
Menopause age, Mean (SD), years	–	–	–	–	–	–	–	–
Age at first live birth, Mean (SD), years	27.6 (4.97)	26.8 (4.93)	26.2 (4.94)	25.2 (5.00)	26.7 (4.97)	26.8 (4.93)	26.8 (5.03)	26.2 (5.14)
Null birth number, N (%)	6150 (26.1%)	4351 (23.9%)	3761 (23.4%)	4726 (27.1%)	3301 (25.0%)	4205 (24.0%)	4905 (24.3%)	6577 (27.0%)
White, N (%)	21706 (92.3%)	16792 (92.1%)	14614 (90.9%)	15597 (89.4%)	11350 (85.9%)	16159 (92.3%)	18828 (93.3%)	22372 (91.8%)
IDM, Mean (SD)	16.5 (13.1)	17.5 (13.6)	18.7 (14.3)	21.9 (15.8)	18.4 (14.1)	17.6 (13.9)	17.8 (13.9)	19.6 (14.9)
BMI, Mean (SD), kg/m2	22.0 (1.79)	24.8 (1.71)	27.6 (2.04)	34.2 (4.91)	22.8 (2.79)	24.3 (3.08)	26.0 (3.63)	31.1 (6.04)
WC, Mean (SD), cm	71.7 (5.35)	78.8 (5.53)	85.6 (6.32)	99.5 (10.8)	73.8 (7.60)	77.5 (8.19)	81.5 (9.23)	92.7 (13.4)
WHR, Mean (SD)	0.77 (0.05)	0.79 (0.06)	0.82 (0.06)	0.85 (0.07)	0.78 (0.06)	0.79 (0.06)	0.80 (0.07)	0.83 (0.07)
Never smoking, N (%)	15229 (64.7%)	11630 (63.8%)	10155 (63.1%)	10930 (62.6%)	8995 (68.0%)	11336 (64.8%)	12743 (63.1%)	14869(61.0%)
Never drinking, N (%)	1638 (7.0%)	1231 (6.8%)	1287 (8.0%)	1846 (10.6%)	1399 (10.6%)	1253 (7.2%)	1347 (6.7%)	2003 (8.2%)
MET, Mean (SD), minutes/week	2750 (2560)	2450 (2340)	2280 (2320)	1970 (2200)	2360 (2350)	2500 (2450)	2480 (2400)	2300 (2370)
Fruit and vegetable intake, Mean (SD), portions/day	2.97 (2.46)	2.82 (2.30)	2.75 (2.38)	2.65 (2.36)	2.72 (2.41)	2.81 (2.37)	2.85 (2.28)	2.83 (2.47)
Family history of cancer, N (%)	6546 (27.8%)	5231 (28.7%)	4839 (30.1%)	5270 (30.2%)	3666 (27.7%)	4945 (28.3%)	6050 (30.0%)	7225 (29.6%)
HRT, N (%)	836 (3.6%)	826 (4.5%)	833 (5.2%)	882 (5.1%)	662 (5.0%)	802 (4.6%)	910 (4.5%)	1003 (4.1%)
Type 2 diabetes, N (%)	372 (1.6%)	360 (2.0%)	456 (2.8%)	1167 (6.7%)	287 (2.2%)	346 (2.0%)	479 (2.4%)	1243 (5.1%)
Hypertension, N (%)	8017 (34.1%)	7794 (42.8%)	8267 (51.4%)	11789 (67.6%)	5367 (40.6%)	7214 (41.2%)	9219 (45.7%)	14067 (57.7%)

Abbreviations: BMI, body mass index; HRT, hormone replacement therapy; IDM, Index of multiple deprivation; WC, waist circumference; WHR, waist to hip ratio.

We documented 1427 cases of the top three gynecologic malignancies (uterine corpus cancer 826 cases, ovarian cancer 533 cases, and cervical cancer 68 cases) over 1613477 person‐years of follow‐up. Tables 2, 3 and Tables S1 illustrate the results of the quartile comparison of traditional anthropometry and body composition. Compared with the first quartile, the fourth quartiles of BMI, WC, and WHR were associated with 2.92 (95% CI 2.16–3.95) times, 2.65 (95% CI 1.97–3.58) times, and 1.67 (95% CI 1.27,2.19) times of higher uterine corpus cancer risk in postmenopausal women, respectively. Regarding body composition, the fourth quartile of WBFM and WBFFM were associated with 2.16 (95% CI 1.49–3.13) times and 1.89 (95% CI 1.31–2.72) times of increased uterine corpus cancer risk, compared to the first quartile. No statistical differences were found in ovarian cancer in the above quartile comparison. We did not include cervical cancer in the quartile analysis as only 68 cases were documented.

**TABLE 2:** Associations between traditional anthropometric measurements and the risk of gynecologic malignancies

		Premenopausal women		Postmenopausal women	
		Case/person‐years	HRs (95% CIs)	Case/person‐years	HRs (95% CIs)
Uterine body cancer	BMI				
	Quartiles				
	Quartile 1	30/152379	Ref	85/251749	Ref
	Quartile 2	30/123564	0.83 (0.40,1.74)	122/280122	1.17 (0.83,1.64)
	Quartile 3	18/109415	0.59 (0.27,1.29)	170/293502	1.43 (1.04,1.99)*
	Quartile 4	58/117359	1.21 (0.60,2.42)	313/285387	2.92 (2.16,3.95)***
	Per kg/m^2^ increase		1.06 (1.02,1.11)*		1.10 (1.08,1.12)***
	WC				
	Quartiles				
	Quartile 1	37/142919	Ref	76/218314	Ref
	Quartile 2	22/139031	0.79 (0.38,1.64)	108/292114	0.92 (0.66,1.31)
	Quartile 3	27/110535	0.63 (0.29,1.33)	174/293312	1.31 (0.95,1.81)
	Quartile 4	50/110232	1.12 (0.57,2.18)	332/307020	2.65 (1.97,3.58)***
	Per cm increase		1.02 (1.00, 1.04)*		1.04 (1.03,1.05)***
Ovarian cancer	BMI				
	Quartiles				
	Quartile 1	30/152379	Ref	94/251749	Ref
	Quartile 2	21/123564	0.63 (0.29,1.38)	110/280122	1.34 (0.95,1.88)
	Quartile 3	19/109415	0.52 (0.26,1.05)	121/293502	1.21 (0.85,1.71)
	Quartile 4	33/117359	0.63 (0.29,1.37)	105/285387	0.97 (0.66, 1.41)
	Per kg/m^2^ increase		0.96 (0.90,1.02)		1.00 (0.97，1.02)
	WC				
	Quartiles				
	Quartile 1	28/142919	Ref	78/218314	Ref
	Quartile 2	24/139031	0.81 (0.36,1.84)	113/292114	1.21 (0.85, 1.73)
	Quartile 3	26/110535	0.74 (0.32,1.70)	118/293312	1.26 (0.88, 1.80)
	Quartile 4	25/110232	0.80 (0.36,1.81)	121/307020	1.04 (0.71,1.53)
	Per cm increase		1.00 (0.98,1.02)		1.00 (0.99,1.01)
Cervix cancer	Per kg/m^2^ increase of BMI		1.02 (0.94,1.11)		0.99 (0.91,1.07)
	Per cm increase of WC		1.01 (0.97,1.05)		1.00 (0.96,1.03)

Note

The HRs (95% CIs) were estimated by multivariate Cox regression models, taking age as the underlying timescale and additionally adjusted for ethnic, index of multiple deprivation, smoking status, alcohol consumption, physical activity, fruit and vegetable intake, diabetes, family history of gynecologic malignancies, surgical history(history of uterus, ovaries, cervical resection), menarche age, oral contraceptives history, reproductie history and height.

Abbreviations: BMI, body mass index; Cis, confidence intervals; HRs, hazard ratios; WC, waist circumference; WHR, waist to hip ratio.

* 0.01≤ *p*‐value <0.05

*** *p*‐value <0.001.

**TABLE 3:** Associations between body composition with the risk of gynecologic malignancies

		Premenopausal women		Postmenopausal women	
		Case/person‐years	HRs (95% CIs)	Case/person‐years	HRs (95% CIs)
Uterine body cancer	WBFM				
	Quartiles				
	Quartile 1	37/154983	Ref	77/244897	Ref
	Quartile 2	23/121826	0.51 (0.23, 1.12)	112/282038	1.17 (0.82,1.67)
	Quartile 3	24/108505	0.70 (0.33,1.48)	191/296142	1.39 (0.98, 1.98)
	Quartile 4	52/117403	0.67 (0.28,1.62)	310/287683	2.16 (1.49,3.13)***
	Per kg increase		1.00 (0.97,1.04)		1.04 (1.02,1.05)***
	WBFFM				
	Quartiles				
	Quartile 1	28/88552	Ref	120/309988	Ref
	Quartile 2	21/116982	0.61 (0.29,1.26)	141/289124	1.24 (0.88,1.75)
	Quartile 3	28/134803	0.68 (0.32,1.45)	163/265310	1.53 (1.08,2.15)*
	Quartile 4	59/162380	1.39 (0.63,3.08)	266/246338	1.89 (1.31,2.72)**
	Per kg increase		1.04 (1.02,1.06)***		1.04 (1.01,1.06)**
Ovarian cancer	WBFM				
	Quartiles				
	Quartile 1	34/154983	Ref	90/244897	Ref
	Quartile 2	13/121826	0.78 (0.35,1.72)	113/282038	1.42 (0.98, 2.06)
	Quartile 3	25/108505	0.46 (0.18,1.18)	110/296142	1.20 (0.82,1.76)
	Quartile 4	31/117403	0.72 (0.26,2.02)	117/287683	1.03 (0.66,1.62)
	Per kg increase		0.99 (0.94,1.03)		1.00 (0.98,1.02)
	WBFFM				
	Quartiles				
	Quartile 1	23/88552	Ref	112/309988	Ref
	Quartile 2	18/116982	0.96 (0.42,2.18)	115/289124	1.22 (0.86,1.73)
	Quartile 3	27/134803	1.03 (0.43,2.47)	102/265310	1.08 (0.74,1.58)
	Quartile 4	35/162380	0.99 (0.35,2.84)	101/246338	1.27 (0.83,1.94)
	Per kg increase		1.01 (0.93,1.10)		1.00 (0.97,1.04)
Cervix cancer	Per kg increase of WBFM		1.02 (0.96,1.09)		1.02 (0.96,1.08)
	Per cm increase of WBFFM		0.96 (0.82,1.11)		0.94 (0.82,1.07)

Note

The HRs (95% CIs) were estimated by multivariate Cox regression models, taking age as the underlying timescale and additionally adjusted for ethnic, index of multiple deprivation, smoking status, alcohol consumption, physical activity, fruit and vegetable intake, diabetes, family history of gynecologic malignancies, surgical history(history of uterus, ovaries, cervical resection), menarche age, oral contraceptives history, reproductie history and height.

Abbreviations: Cis, confidence intervals; HRs, hazard ratios; WBFFM, whole body fat‐free mass; WBFM, whole body fat mass.

* 0.01 ≤ *p*‐value <0.05

** 0.001 ≤ *p*‐value <0.01

*** *p*‐value <0.001.

We further investigate the risk of the major gynecologic malignancies with continuous per unit increase in anthropometric measurements and FM/FFM. In multivariable analysis, BMI, WC, and WHR were positively associated with the risk of uterine corpus cancer in both premenopausal (Adjusted HR per unit increase: BMI 1.06, 95% CI 1.02–1.11; WC: 1.02 95% CI 1.00–1.04; WHR 1.01, 95% CI 0.97–1.04) and postmenopausal women (Adjusted HR per unit increase: BMI 1.10, 95% CI 1.08–1.12; WC 1.04, 95% CI 1.03–1.05; WHR 1.03, 95% CI 1.02–1.05) (Table 2 and Table S1). There is no sufficient evidence of associations between BMI/WC/WHR and risk of ovarian or cervical cancer in pre or post menopause women.

**FIGURE 1:**
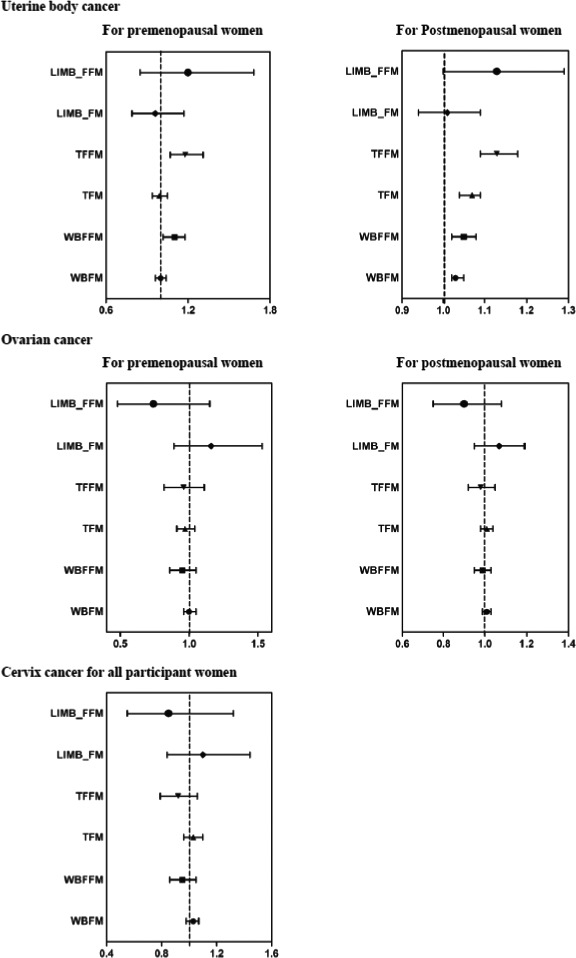
Associations between body fat distribution and risk of gynecologic malignancies. The HRs (95% CIs) were estimated by multivariate Cox regression models, taking age as the underlying timescale and additionally adjusted for ethnic, index of multiple deprivation, smoking status, alcohol consumption, physical activity, fruit and vegetable intake, diabetes, family history of gynecologic malignancies, surgical history(history of uterus, ovaries, cervical resection), menarche age, oral contraceptives history, reproductie history and height. Abbreviations: Cis, confidence intervals; HRs, hazard ratios; LIMB_FM, limb fat mass; LIMB_FFM, limb fat‐free mass; TFM, trunk fat mass; TFFM, trunk fat‐free mass; WBFM, whole body fat mass; WBFFM, whole body fat‐free mass

As for the associations between continuous body composition measurements and gynecological malignancies, WBFM/WBFFM presented statistically associations with increased risk of uterine corpus cancer but were not associated with the other two gynecological malignancies (Table 3 and Figure 1). For premenopausal women, continuous trend in WBFFM were associated with an increased risk of uterine corpus cancer (Adjusted HR per unit increase 1.04, 95% CI 1.02–1.06). For postmenopausal women, both WBFFM and WBFM presented positive associations with increased risk of uterine corpus cancer. The results were generally unchanged in other sensitivity analyses by lagging the exposure for a time window of 2 years, applying the complete case analysis (Table S2). We further evaluated the risk of major gynecological malignancies according to the distribution of FM/FFM, and result showed that body fat distribution was only statistically associated with uterine corpus cancer (Figure 1). For premenopausal women, FFM distributed in the trunk (TFFM) was associated with an increased risk of uterine corpus cancer (Adjusted HR per unit increase 1.18, 95% CI 1.07–1.31). FFM and FM distributed in the trunk were associated with 1.13 (95% CI 1.09–1.18) times and 1.07 (95% CI 1.04–1.09) times of increased uterine corpus cancer risk in postmenopausal women.

Assessment of the nonlinear association of body composition with gynecological malignancies showed that uterine corpus cancer risk was associated with higher WBFFM and TFFM in premenopausal women, and higher WBFM, WBFFM, TFFM, and trunk fat mass (TFM) in postmenopausal women. FM/FFM distributed in the limbs present U‐shaped associations with substantially increased risk of uterine corpus cancer in postmenopausal women (Figures S1–S3).

## 4 DISCUSSION

This large prospective analysis of 245,009 female participants shows that traditional anthropometric indicators such as BMI, WC, and WHR, might be associated with an increased risk of uterine corpus cancer. This trend tends to be stronger in postmenopausal women. The above results are generally consistent with previous prospective studies.^10^, ^15^ Our study did not find sufficient evidence of associations between BMI/WC/WHR and risk of ovarian or cervical cancer. The link between obesity and ovarian/cervical cancer is itself controversial, with more evidence at present tends not to support obesity as a direct risk factor for them.^8^, ^16^

Obesity, especially abdominal adiposity, has been considered as a risk factor for certain hormone‐dependent tumors in women, such as breast cancer and endometrial cancer. As an indicator of overall adiposity, BMI is unable to represent the whole picture of obesity. Whether WC, WHR and other measures reflecting abdominal adiposity are more advantageous than BMI in reflecting the risk of malignant tumors is still inconclusive.^17^, ^18^ Body composition may present a more precise measure of overall fatness and fat distribution than traditional measures and provide further information on metabolic capacity and load. A recent study found that body composition is a better predictor of invasive breast cancer risk in postmenopausal women than BMI.^19^ Additionally, there are also ethnic differences in body composition. For instance, Asians have higher body fat percentage(BFP), more belly fat deposits and lower muscle mass for a given BMI compared with Caucasians.^20^ Further investigation of the relationship between body composition and the risk of malignancies in a more broadly representative population is still required.

Current epidemiological studies evaluating the associations between body composition and gynecological malignancies risk remain inadequate. A cohort study involving 18,700 women found that WBFM was associated with increased risk of ovarian cancer (HR per 10 kg 1.23, 95% CI 1.01–1.49).^21^ However, this study is limited as the number of participants in the cohort is small and some confounding factors (such as diet) were not adjusted. So far, there is no cohort study on the relationship between body composition and the risk of cervical cancer. A cross‐sectional survey of 20,236 women shows a significant association between BFP and cervical cancer (OR 1.027, 95% CI 1.006–1.048),^22^ and this epidemiological association is higher as the BFP increases. However, just like BMI and WC, we do not observe associations between body composition and ovarian/cervical cancer. A recent paper, which also based on the UK Biobank cohort study, reported that BFP and WBFM were positively associated with the risk of endometrial cancer in postmenopausal women.^10^ In addition to above studies, we also find that WBFFM and TFFM are risk factors for uterine corpus cancer in both premenopausal and postmenopausal women, which is not consistent with common sense. FFM mainly contains the contents of muscle, viscera, and bone, which is often considered an indirect indicator of muscle mass.^23^ It has been believed that a greater FFM, and therefore, a greater resting metabolic rate, will protect against obesity and associated comorbidities.^24^ But in our study, FFM is likely to be predictor of uterine corpus cancer risk in both premenopausal or postmenopausal women. Similar findings have also shown in previous studies evaluating body composition and risk of breast cancer,^25^ prostate cancer,^26^ rectal cancer,^27^ and lung cancer.^28^ The results of our team's unpublished research also show that FFM tended to associate with increased risk of gastric cancer in both genders (Males: HR 1.70, 95% CI 1.01–2.89; Females: HR 2.47, 95% CI 1.15–5.32).

The exact mechanisms of FFM on uterine corpus cancer risk remain unclear. A potential explanation is that FFM is associated with nutritional factors, such as red meat consumption, which is linked with increased risk of uterine corpus cancer.^29^ A recent study has found that a large FFM is a predictor of insulin resistance (IR) independently of the aging process.^30^ Meanwhile, IR plays a central role in endometrial cancer, which accounts for majority of uterine corpus cancer.^31^ So another possible pathogenesis is that FFM increases IR, which in turn increases the risk of uterine corpus cancer. As ethnic differences in body composition also confer Asians more likely to develop IR than Caucasians with the same BMI.^32^ Therefore, the relationship between FFM and IR and their comorbidities should be further elucidated in different ethnic groups to clarify the potential mechanism underlying the relationship between FFM and uterine corpus cancer.

### 4.1 Strength and limitations

To the best of our knowledge, the present study is the first cohort study evaluating body composition and the major gynecologic malignancies risk. This analysis was based on a well established nationwide cohort of over 0.45 million participants, with detailed measurement of body composition and a wide range of known and putative gynecologic malignancies risk factors, allowing us to adequately control potential confounding factors. For the first time, we evaluated the effect of FFM on uterine corpus cancer risk. In addition, we applied a range of approaches to evaluate body composition effects, including the assessment of nonlinearity and effect modification. Lastly, a wide range of robust sensitivity analyses further strengthened our confidence in the results.

This study has limitations. First, as an observational study, we cannot eliminate the residual confounding effect and confirm the causal relationship. Second, with limited cases of other types of uterine corpus cancer (except for endometrial cancer) according to the ICD‐10 code in UK Biobank and absence of histological classification data, we could not separately evaluate associations of body composition measurements with subtype of uterine corpus cancer. Further studies investigating these associations are still required in the future. Third, we only documented 68 cases of cervical cancer in UK Biobank database, thus we were unable to evaluate the associations of body composition and cervical cancer risks separately by pre/postmenopausal status. However, previous studies have demonstrated that HPV infection is the most important risk factor for cervical cancer, and the change of sex hormone before and after menopause might have no effect on the incidence of cervical cancer^[2]^. Fourth, body composition measured by bioelectrical impedance analysis in this study may be influenced by other factors such as ethnicity, phase of menstrual cycle, and underlying medical conditions. However, high correlation between bio‐impedance and the DXA‐derived measures indicated that bio‐impedance is reliable. Last, as most participants in the UK Biobank were of European ancestry, the generalizability of the study findings to other ethnicities remains unclear.

## 5 CONCLUSION

Overall, this large‐scale prospective study suggests that FFM is associated with an increased risk of uterine corpus cancer in both premenopausal and postmenopausal women. We did not observe associations between body composition measures and ovarian/cervical cancer. In clinical and public health practice, our findings provided evidence for individualized weight management for the prevention of gynecologic malignancies. Interventions for controlling excessive lean mass may have benefits in reducing uterine corpus cancer. Further research is warranted to confirm the causality and to investigate the underline mechanism of the effects of FFM on uterine corpus cancer.

## CONFLICT OF INTEREST

The authors declared that they have no conflicts of interest to this work.

## ETHICAL STATEMENT

UK Biobank was approved by the North West Multicentre Research Ethics Committee (MREC), the Patient Information Advisory Group (PIAG) in England and Wales, and the Community Health Index Advisory Group in Scotland (CHIAG).

## Supporting information

Supplementary Material

## Data Availability

The data used to support the findings of this study are available from the corresponding author upon request.
