# Supplementary material for: Body composition and risk of major gynecologic malignancies: Results from the UK Biobank prospective cohort
Source: Cancer Med. 2021 Jun 10;10(13):4522–31. doi: 10.1002/cam4.3925 (PMC8267135; doi:10.1002/cam4.3925)
Supplement: Supplementary file 1 — Supplementary Material [file CAM4-10-4522-s001.docx]

| **Premenopausal women** |  |  |  |
| --- | --- | --- | --- |
| 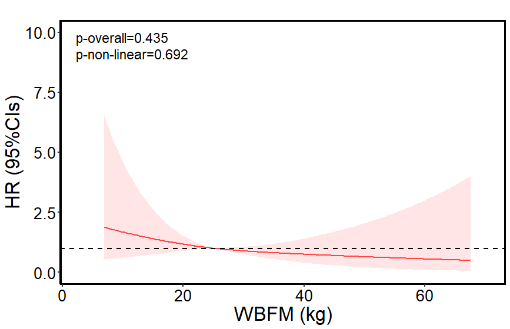 | 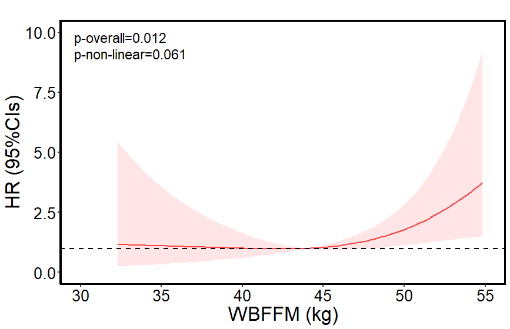 | 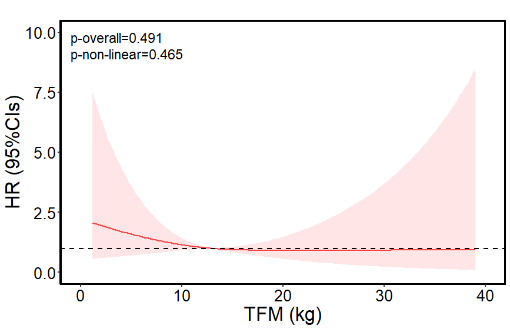 | 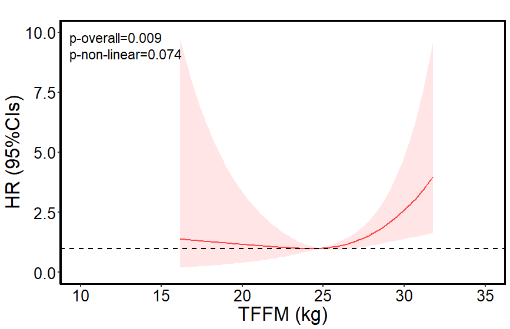 |
| 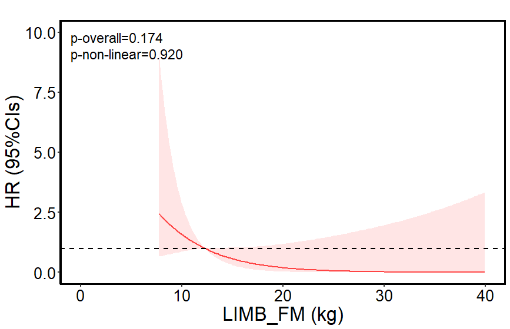 | 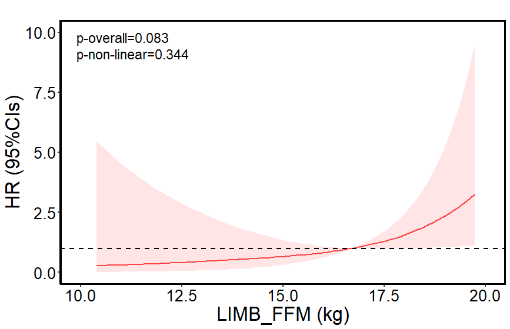 | 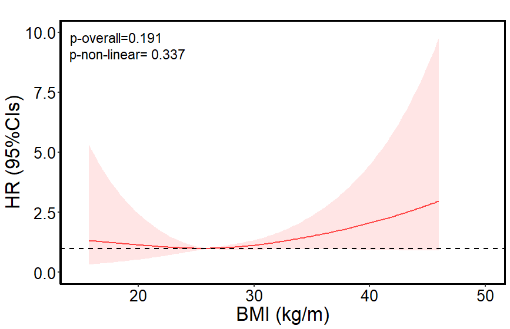 | 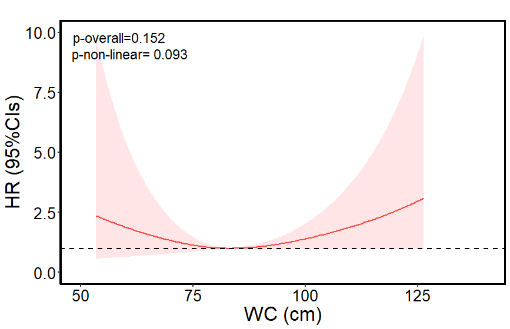 |
| **Postmenopausal women** |  |  |  |
| 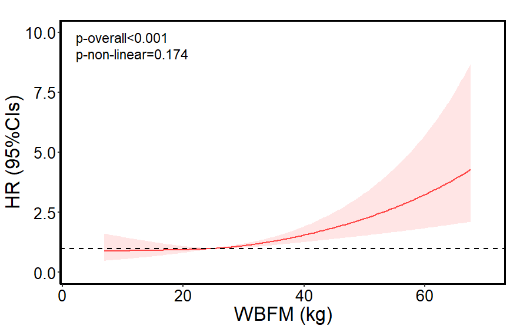 | 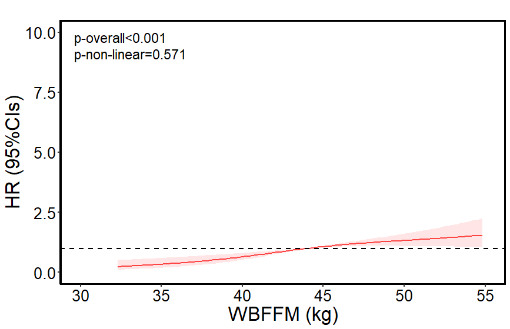 | 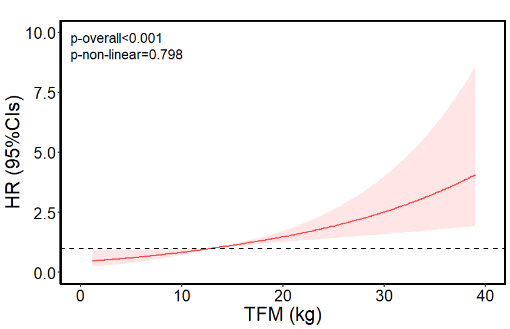 | 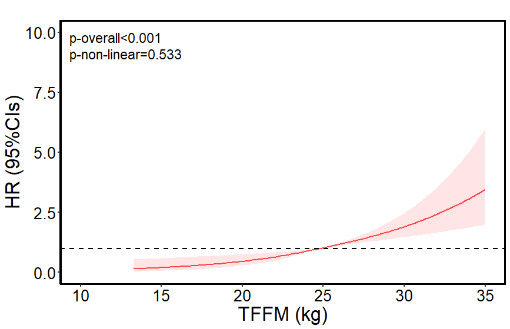 |
| 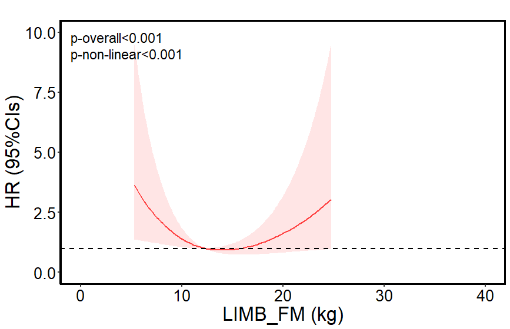 | 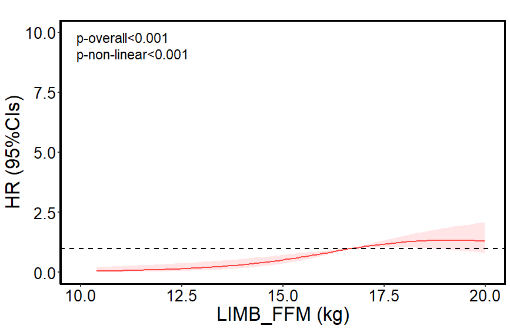 | 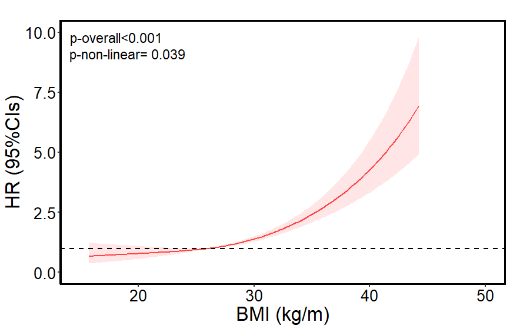 | 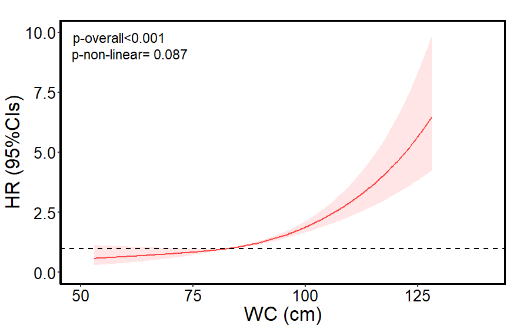 |

Fig S1. Associations between anthropometrics indicators/body fat distribution and risk of uterine corpus cancer, allowing for non-linear effects.

| **Premenopausal women** |  |  |  |
| --- | --- | --- | --- |
| 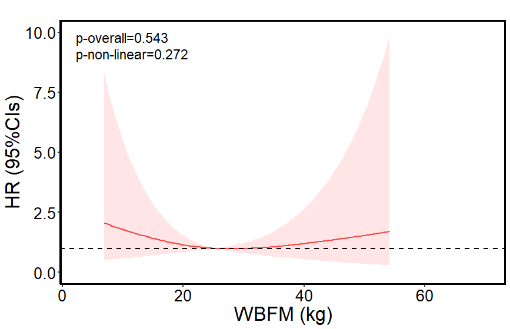 | 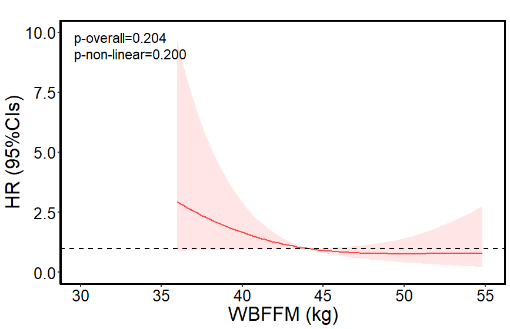 | 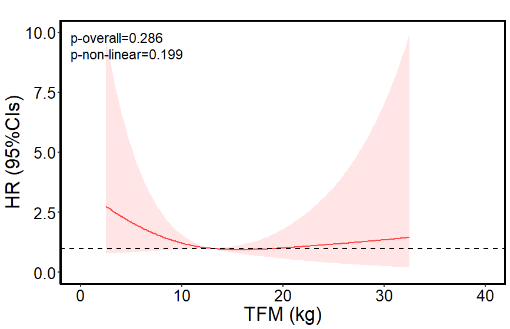 | 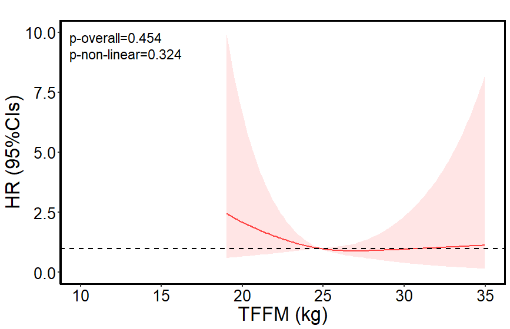 |
| 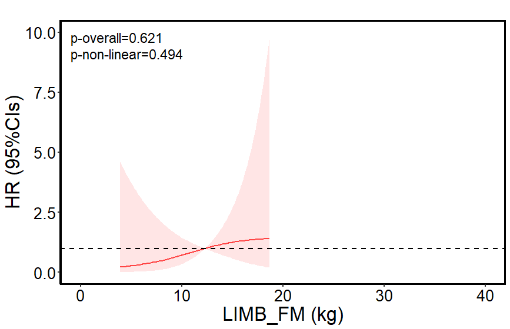 | 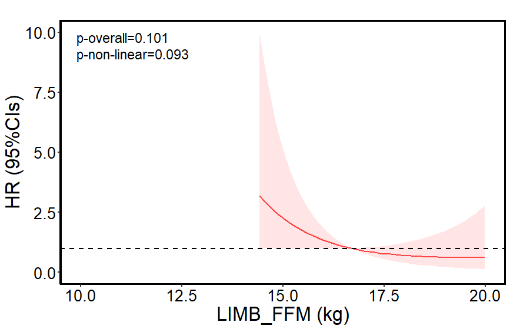 | 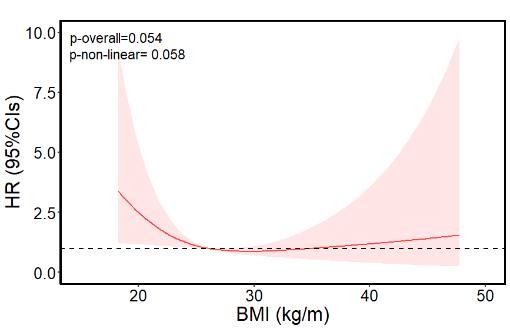 | 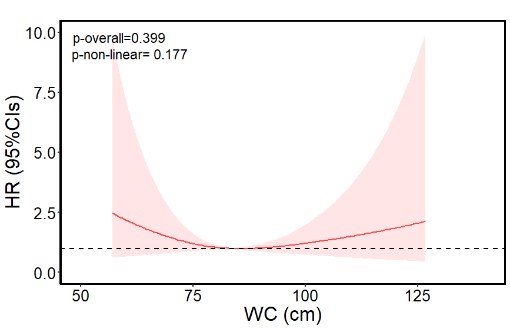 |
| **Postmenopausal women** |  |  |  |
| 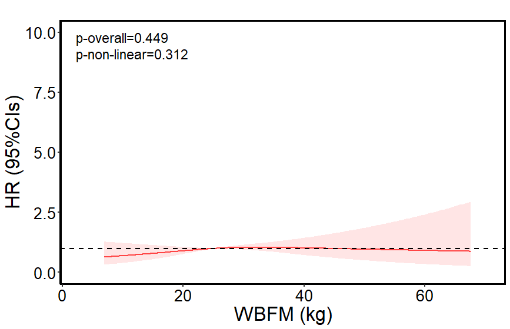 | 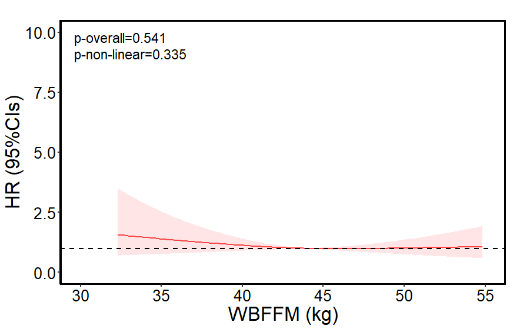 | 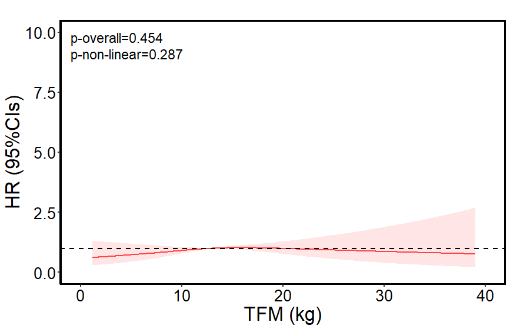 | 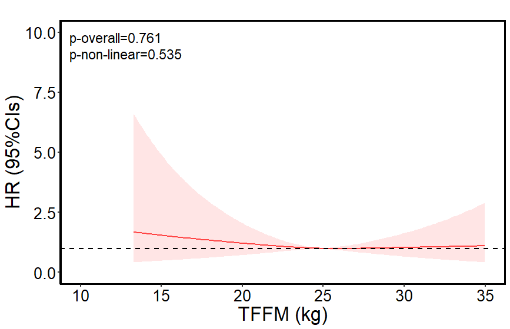 |
| 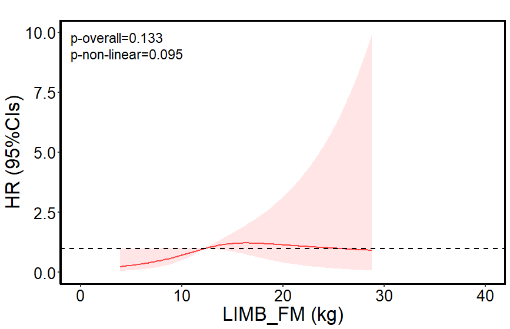 | 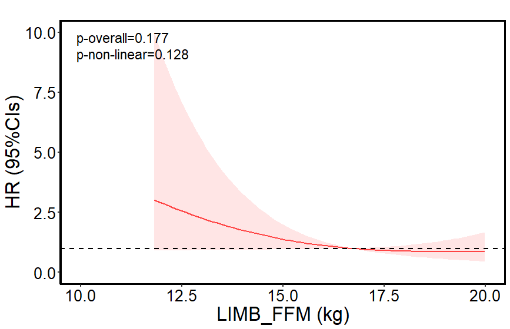 | 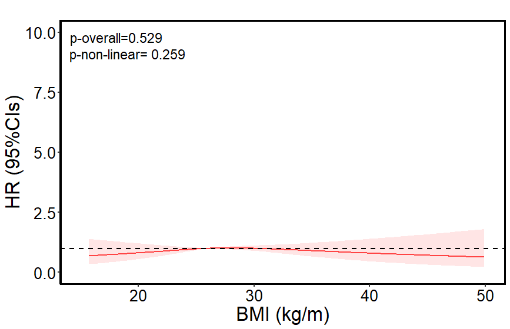 | 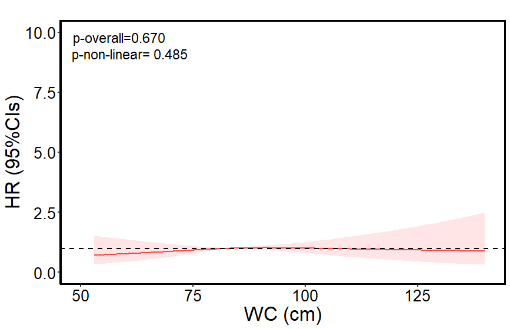 |

Fig S2. Associations between anthropometrics indicators/body fat distribution and risk of ovary cancer, allowing for non-linear effects.

| 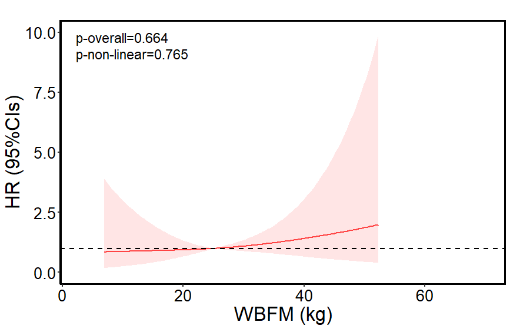 | 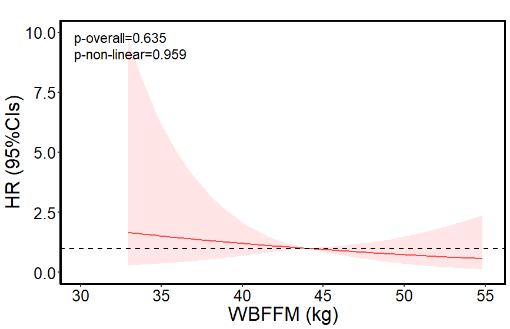 | 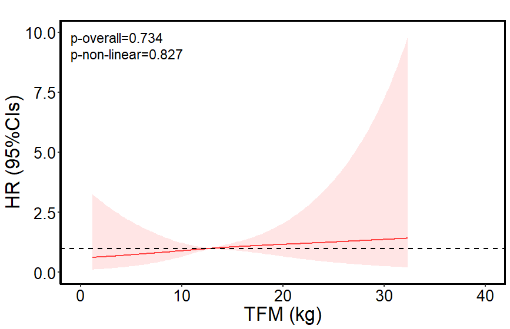 | 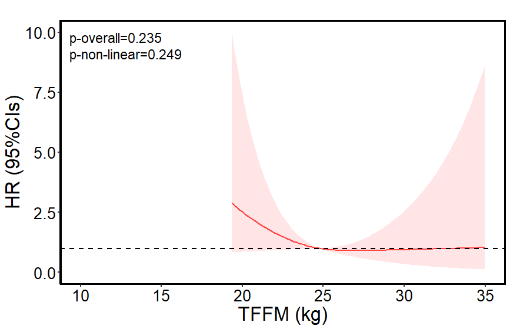 |
| --- | --- | --- | --- |
| 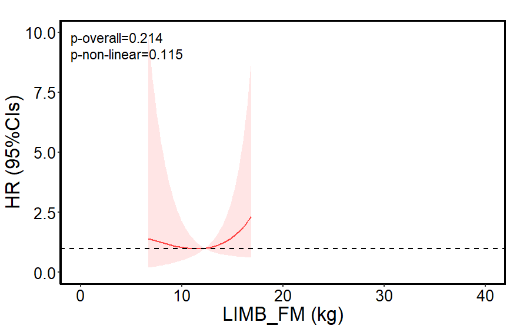 | 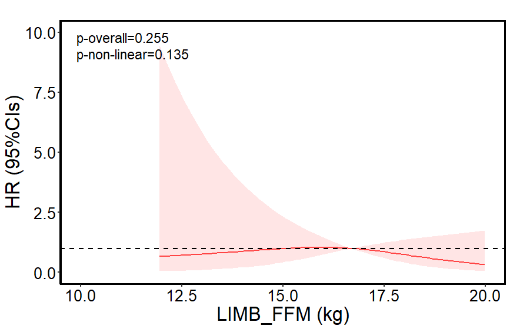 | 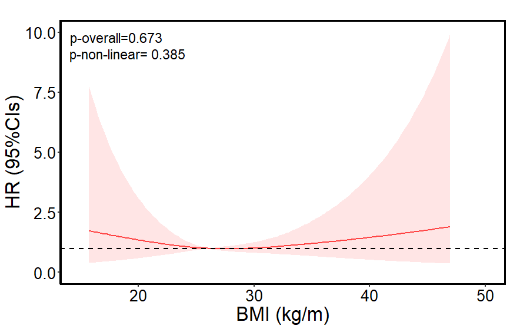 | 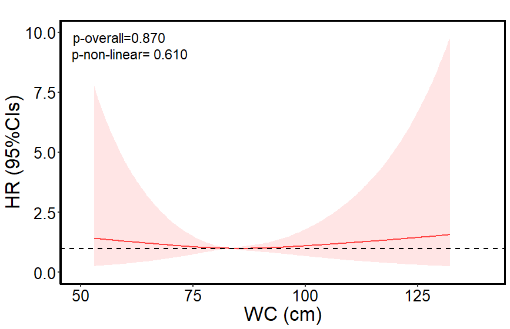 |

Fig S3. Associations between anthropometrics indicators/body fat distribution and risk of cervix cancer, allowing for non-linear effects.

**Table S1.** Estimated HRs (95% CIs) for the associations between WHR with the risk of gynecologic malignancies

|  |  | **Premenopausal women** | |  | **Postmenopausal women** | | | |
| --- | --- | --- | --- | --- | --- | --- | --- | --- |
|  |  | **Case/person-years** | **HRs (95% CIs)** | | | **Case/person-years** | | **HRs (95% CIs)** |
| **Uterine body cancer** | **Quartiles** |  |  |  |  | |  | |
|  | Quartile 1 | 34/161139 | Ref |  | 102/243226 | | Ref | |
|  | Quartile 2 | 41/133538 | 0.60 (0.29,1.27) |  | 139/273423 | | 0.99 (0.73,1.33) | |
|  | Quartile 3 | 24/112736 | 0.83 (0.43,1.62) |  | 169/289969 | | 1.02 (0.76,1.37) | |
|  | Quartile 4 | 37/95304 | 0.76 (0.39,1.48) |  | 280/304142 | | 1.67 (1.27,2.19)*** | |
|  | Per unit increase | | 1.01 (0.97,1.04) |  |  | | 1.03 (1.02,1.05)*** | |
| **Ovarian cancer** | **Quartiles** |  |  |  |  | |  | |
|  | Quartile 1 | 30/161139 | Ref |  | 96/243226 | | Ref | |
|  | Quartile 2 | 27/133538 | 0.54 (0.21,1.36) |  | 114/273423 | | 1.22 (0.87,1.71) | |
|  | Quartile 3 | 27/112736 | 1.10 (0.51,2.34) |  | 97/289969 | | 0.84 (0.59,1.22) | |
|  | Quartile 4 | 19/95304 | 0.72 (0.31,1.66) |  | 123/304142 | | 1.16 (0.82,1.63) | |
|  | **Per unit increase** | | 0.99 (0.95,1.04) |  |  | | 1.00 (0.98,1.02) | |
| **Cervix cancer** | **Per unit increase** | | 1.03 (0.97,1.1) |  |  | | 1.00 (0.94,1.05) | |

^#^ The HRs (95%CIs) were estimated by multivariate Cox regression models, taking age as the underlying timescale and additionally adjusted for ethnic, index of multiple deprivation, smoking status, alcohol consumption, physical activity, fruit and vegetable intake, diabetes, family history of gynecologic malignancies, surgical history(history of uterus, ovaries, cervical resection), menarche age, oral contraceptives history, reproductie history and height.

*0.01≤ *P-value* < 0.05, ** 0.001≤ *P-value* < 0.01, ****P-value* < 0.001;

Abbreviations: Cis, confidence intervals; HRs, hazard ratios; WHR, waist to hip ratio.

**Table S2** Sensitive analyses

|  | Sensitive analysis 1 [HRs (95%CIs)] ^a^ | | Sensitive analysis 2 [HRs (95%CIs)] ^b^ | |
| --- | --- | --- | --- | --- |
|  | Model 1^c^ | Model 2 ^d^ | Model 1 ^c^ | Model 2 ^d^ |
| Premenopausal women |  |  |  |  |
| Uterine body cancer |  |  |  |  |
| WBFM | 1.03 (1.01,1.06)** | 1.01 (0.97,1.05) | 1.03 (1,1.05) | 0.99 (0.96,1.03) |
| WBFFM | 1.01 (0.96,1.07) | 1.04 (0.97,1.12) | 1.04 (0.99,1.09) | 1.09 (1.02,1.17)* |
| Ovarian cancer |  |  |  |  |
| WBFM | 1.01 (0.98,1.04) | 1.00 (0.95,1.05) | 1.02 (0.99,1.05) | 1.00 (0.95,1.05) |
| WBFFM | 1.02 (0.95,1.08) | 0.99 (0.9,1.09) | 0.99 (0.93,1.05) | 0.99 (0.91,1.09) |
| Cervix cancer |  |  |  |  |
| WBFM | 0.98 (0.91,1.06) | 0.99 (0.90,1.1) | 1 (0.94,1.05) | 1.01 (0.95,1.08) |
| WBFFM | 1.02 (0.88,1.18) | 1.01 (0.83,1.24) | 1.03 (0.92,1.16) | 1.01 (0.88,1.16) |
| Postmenopausal women |  |  |  |  |
| Uterine body cancer |  |  |  |  |
| WBFM | 1.04 (1.03,1.05)*** | 1.04 (1.02,1.06)*** | 1.04 (1.03,1.05)*** | 1.03 (1.02,1.05)*** |
| WBFFM | 1.03 (1.01,1.06)** | 1.03 (1.00,1.06)* | 1.03 (1,1.05) | 1.04 (1.01,1.07)* |
| Ovarian cancer |  |  |  |  |
| WBFM | 1 (0.99,1.02) | 1.01 (0.98,1.03) | 1.00 (0.99,1.02) | 1.00 (0.98,1.02) |
| WBFFM | 1 (0.97,1.03) | 0.99 (0.95,1.04) | 1.01 (0.98,1.04) | 1.01 (0.97,1.06) |
| Cervix cancer |  |  |  |  |
| WBFM | 1 (0.95,1.06) | 1.02 (0.95,1.09) | 1.02 (0.97,1.07) | 1.04 (0.97,1.12) |
| WBFFM | 0.98 (0.88,1.09) | 0.96 (0.84,1.11) | 0.94 (0.84,1.04) | 0.89 (0.77,1.03) |

^a^ Sensitive analysis 1: lagging the exposure for 2 years and allow a time window for gynecologic malignancies risks;

^b^ Sensitive analysis 2: the complete-case analysis was applied to verify the influence of missing data.

^c^ Model 1: minimally cox regression models, only taking age as the underlying timescale.

^d^ Model 2: multivariate Cox regression models, taking age as the underlying timescale and additionally adjusted for ethnic, index of multiple deprivation, smoking status, alcohol consumption, physical activity, fruit and vegetable intake, diabetes, family history of gynecologic malignancies, surgical history(history of uterus, ovaries, cervical resection), menarche age, oral contraceptives history, reproductie history and height.

^*^0.01≤ *P*-value < 0.05, ^**^ 0.001≤ *P*-value < 0.01, ^***^*P*-value < 0.001;

Abbreviations: Cis, confidence intervals; HRs, hazard ratios; WBFM, whole body fat mass; WBFFM, whole body fat-free mass
